# Criminal Abortion

**Published:** 1867

**Authors:** 


*Criminal Abortion.*—This subject, which has for a long time awakened professional attention, and has been urged upon the public conscience in all the modes of remonstrance, has of late come up in an original and, the JOURNAL is half-disposed to say, a *questionable* shape. The *Northwestern Christian Advocate*, the powerful organ of the great Methodist denomination, has taken up the topic, and devoted an entire page to its discussion. It seems to us a dangerous course to pursue, but we are not prepared to condemn it. The evil is clearly a great one, but like other evils it is liable to be both over and under estimated. Whether more good or injury will result from bringing the crime openly before both the criminal and pure minded, is a question for casuists to determine. Thousands will thus learn for the first time the comparative facility of the act, whilst they will give no heed to the moral aspect of the thing. Of the physical evils they will take their chances. Every physician will admit having been occasionally called upon to assist in the misdeed, but the parties infallibly have some excuse which to them is a healing plaster to the sore offence.

The most sacred relations of life are being dragged to public notoriety in lectures, in published books, and broadcast newspapers. The adolescent mind is corrupted by ideas of nameless crimes, gathered largely from the advertising columns of newspapers, even of the, so-called, religious press. This article, questionable as it appears to us, we trust may have the effect, at least, to turn attention to, and cause the utter suppression of, that class of newspaper publications which pander to depraved minds under cover of affording relief to debauched bodies.

It occurs to the JOURNAL that, outside of strictly professional publications, this subject should be developed only in private conversation with patients; or such tractates as the "Why Not?" of Prof. STORER, be placed in the hands of such married people as the judicious physician, or perhaps religious pastor, might deem likely to be benefited by the perusal. Half grown boys and girls are not likely to be benefited by this, to them, curious and entertaining literature.

The JOURNAL will recur to this subject hereafter, but, meanwhile, puts on record its opinion that the apparent diminution of fecundity, appealed to as evidence of the great prevalence of this crime, is not due to the direct procuring of abortion, but to the practice of onanism, and the use of mechanical preventives—advertisements of which boldly stare out of the newspapers, (even some of the religious sort), and which are shamelessly taught in "Lectures to Gentlemen Only" and "Lectures to Ladies Only," and the utterances of which are listened to by even some of the elect.

The JOURNAL puts also on record its belief that, were professional physicians called to testify in court, they would be unanimous in the statement that they are far oftener called upon to remedy sterility, or barrenness, than to obviate fertility.

The JOURNAL also declares its conviction that the procuring of abortions criminally, save in the smallest conceivable proportion of instances, is carried on, outside the pale of the legitimate medical body, by a brood of quacks and pretenders that members of the reverend clerical profession have done more than any and all others to foster and sustain.

We are the more happy to see their chief means of support smitten by a similar power, although we believe the skirts of the *Advocate* are most uncommonly clear from this latter *peccadillo.*

